# Recombinant Human Thyrotropin-Aided Radioiodine Therapy in Patients with Metastatic Differentiated Thyroid Carcinoma

**DOI:** 10.1155/2012/670180

**Published:** 2011-08-18

**Authors:** Ivana Zagar, Andreja A. Schwarzbartl-Pevec, Barbara Vidergar-Kralj, Rika Horvat, Nikola Besic

**Affiliations:** ^1^Department of Nuclear Medicine, Institute of Oncology, 1000 Ljubljana, Slovenia; ^2^Department of Surgery, Institute of Oncology, 1000 Ljubljana, Slovenia

## Abstract

Our aim was to test the efficacy of 131-I therapy (RIT) using recombinant human TSH (rhTSH) in patients with differentiated thyroid carcinoma (DTC) in whom endogenous TSH stimulation was not an option due to the poor patient's physical condition or due to the disease progression during L-thyroxin withdrawal. The study comprised 18 patients, who already have undergone total or near-total thyroidectomy and radioiodine ablation and 0–12 (median 5) RITs after L-thyroxin withdrawal. Our patients received altogether 44 RITs using rhTSH while on L-thyroxin. Six to 12 months after the first rhTSH-aided RIT, PR and SD was achieved in 3/18 (17%) and 4/18 patients (22%), respectively. In most patients (*n* = 12; 61%) disease progressed despite rhTSH-aided RITs. As a conclusion, rhTSH-aided RIT proved to add some therapeutic benefit in 39% our patients with metastatic DTC, who otherwise could not be efficiently treated with RIT.

## 1. Introduction

In thyroidectomized patients with differentiated thyroid carcinoma (DTC), it is believed that serum TSH >30 mU/L optimizes radioiodine trapping and retention and thyroglobulin (Tg) synthesis by neoplastic cells and is therefore necessary for reliable serum Tg testing and efficacious radioiodine therapy [1–4]. Such TSH elevation traditionally has been achieved by a 4–6-week withdrawal of L-thyroxin (LT4) suppression therapy. 

Thyroid hormone withdrawal (THW), however, generally causes clinical hypothyroidism which is associated with physical and emotional discomfort, cognitive dysfunction, and impaired quality of life and ability to work in most patients. Clinical hypothyroidism is especially poorly tolerated by elderly patients with serious concomitant diseases, to whom it may pose an important health risk [5–8]. In addition, given its weeks-long duration and the additional time required for thyroid hormone concentrations to return to suppressive levels after LT4 therapy is restarted [9, 10], THW entails a protracted period of serum TSH elevation. In patients with metastases in confined spaces such as the brain, spine, or airways or with a high tumor burden, protracted TSH elevation sometimes has been documented to stimulate tumor expansion or growth, causing compressive or obstructive symptoms or worsening DTC prognosis [11].

Recombinant human TSH (rhTSH) was developed to provide TSH stimulation without THW and the morbidity secondary to clinical hypothyroidism [6, 7]. Because rhTSH is administered in a course of two consecutive daily injections and because of the relatively short half-life of TSH, rhTSH use results in TSH elevation lasting only days. In theory, rhTSH use therefore could decrease the risk of tumor stimulation and its sequelae. 

RhTSH has been approved since 1998 in the United States and 2000 in Europe as an adjunct to diagnostic and follow-up procedures in patients with DTC [11–15]. In 2005, the drug was approved in Europe to aid in the administration of large activities of 131-iodine (131-I) for postsurgical remnant ablation in patients with DTC [16].

RhTSH-aided radioiodine treatment (RIT) of patients with inoperable locally aggressive or distant metastatic DTC, is, however, still considered experimental [14, 17]. Although as of August, 2004 some 216 cases of such RIT had been published [14], these reports varied greatly in their level of detail, particularly with respect to outcome, and only one published series [18] numbered more than 15 patients. Moreover, only a single case of rhTSH-aided RIT of Hürthle cell carcinoma appears to have been published [19]. 

We sought to test the safety and efficacy of rhTSH as an alternative to THW in stimulating RIT of patients with advanced DTC. Therefore, from 2002–2007, we gave 44 rhTSH-aided RITs to 18 such patients. These patients predominantly were elderly and had concomitant disease(s), history of severe hypothyroid or compressive symptoms or evidence of tumor progression during THW, or both, which precluded THW. Of note, based upon our data suggesting that RIT may be effective in patients with histologically confirmed Hürthle cell carcinoma [20, 21], our series included six such patients. We now report on our initial experience with rhTSH-aided RIT.

## 2. Patients and Methods

### 2.1. Patients and Ethical Considerations

The present series comprises 18 patients (12 females, 6 males) who received rhTSH-aided RIT for histologically confirmed, advanced recurrent or residual DTC at our tertiary referral clinic, the Institute of Oncology, Ljubljana, Slovenia from January 2002 through September 2007. The patients were followed for a median, 50 months (range, 15–99 months) after rhTSH-aided RIT. Earlier, more limited data on patients number 2, 3, 4 and 6 have been published elsewhere [20].

Table 1 includes patient characteristics for the series. The median age was 72 years (range, 37–83 years) and 13 (72%) patients were over 65. Five (28%) patients had papillary carcinoma, of whom two had the follicular variant and one had poorly differentiated tumor, seven (39%) had follicular carcinoma, of whom one had the insular variant, and one poorly differentiated tumor, and six (33%) patients had Hürthle cell carcinoma.

The primary diagnosis was established from 1993–2003. At the initial presentation at our clinic (“presentation”), pT stage [22] according to the tumor node metastasis classification [16] was pT4 in 12 patients (67%), pT3 in five patients (28%) and pT2 in one patient (5%). N stage was N0 in 14 (83%) patients, and N1 in three patients (17%). Distant metastases were evident at presentation in eight (44%) patients. At the time of the first rhTSH-aided RIT, the metastatic site was lungs in five patients (27.5%), bone + lungs in four patients (22%), only bone in three patients (16.5%), mediastinum in two patients (11%), bone + brain, bone + liver, bone + mediastinum, and lung + kidney were metastatic sites in one patient (5.5%). In two patients (11%) distant metastases were found as well as regional recurrent tumor which was not surgically treated (one was inoperable, while in the other patient numerous metastatic sites were present). 

**Figure 1:**
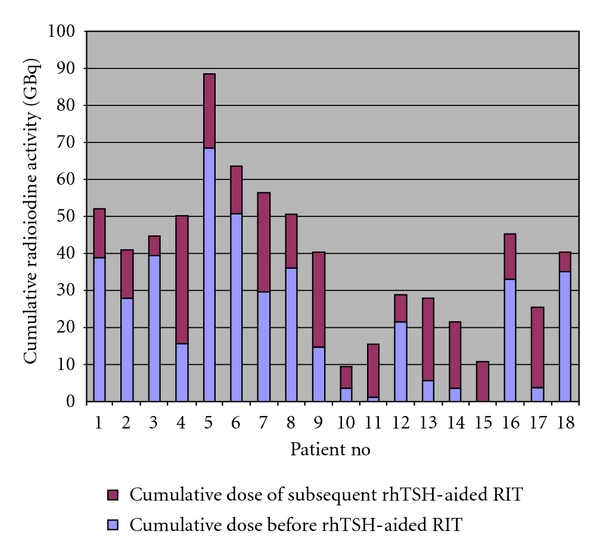
Cumulative radioiodine activity per patient by TSH stimulation method.

All patients had total or near-total thyroidectomy, and all but one had previous RIT (median: 5 treatments, range: 1–12 treatments after THW, with cumulative activities ranging from 3.6 to 68.33 GBq (median 27.89 GBq) as shown on Figure 1. The time from the most recent prior THW-aided RIT to referral for rhTSH-aided RIT ranged from 2.5 to 84 months (median: 12 months). Twelve (67%) patients had undergone external beam radiotherapy (EBR) within 1–120 months (median: 6.5 months) before referral (another three patients after the first rhTSH-aided RIT), while eight (44%) had chemotherapy with either adriamycin or vinblastine from within one week to 120 months (median: 6 months) before referral.

Specific indications for rhTSH were generally multiple, including one or more of (A) concomitant disease (*n* = 5) or (B) metastasis-related neurological symptomatology (*n* = 9) that posed a danger of important exacerbation under hypothyroid conditions or (C) a history of severe symptoms, evidence of tumor progression, for example, an immense increase in serum Tg, or both during hypothyroidism (*n* = 4) or (D) frailty due to advanced age (*n* = 10). Our main aim in giving rhTSH, however, was to avoid the risk of tumor progression with concomitant symptoms, related to that. 

The study was approved by our center's Institutional Review Board and Ethics Committee and performed in accordance with the ethical standards of the World medical association declaration of Helsinki. Written informed consent was obtained from every patient before each rhTSH-aided RIT.

### 2.2. RhTSH-Aided RIT and Follow-Up Protocol

**Figure 2:**
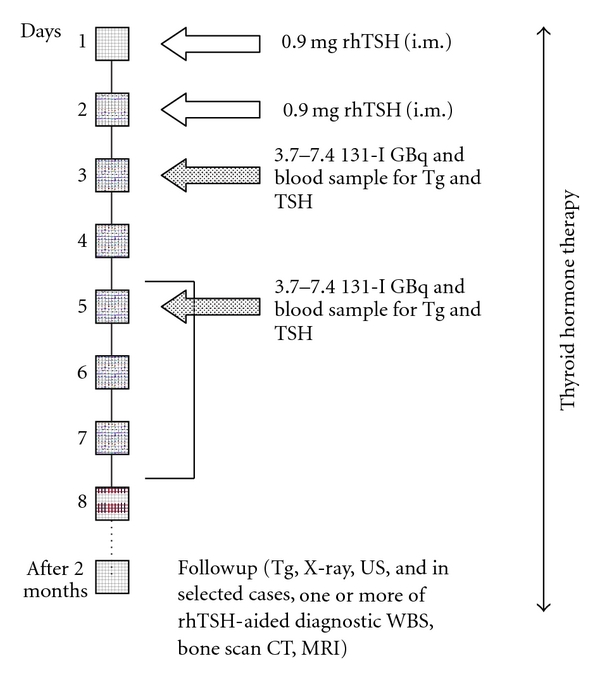
Protocol for rhTSH-aided radioiodine treatment and follow-up.

Figure 2 illustrates our rhTSH-aided RIT and follow-up protocol. For each RIT, patients received an intramuscular injection of 0.9 mg rhTSH (Thyrogen, Genzyme Corporation, Cambridge, Mass, USA) on two consecutive days, followed on the third day by the oral administration of a capsule of 5.5–7.4 GBq of 131-I. RIT activities were determined empirically, according to the same standard institutional criteria as for activities after THW. No adjustment was made in the RIT activity to offset the faster renal clearance when patients are euthyroid [23]. Dosimetry of metastases was not done because it is not routinely performed at our department.

Decision when to give multiple rhTSH-aided RITs was tailored individually. We stopped with rhTSH-aided RITs at cumulative dose of approximately 37 GBq, although some of the patients were heavily pretreated or when rhTSH-aided RITs proved ineffective.

In all patients, a low-iodine diet was prescribed for approximately two weeks preceding the radioiodine administration, and at the time of that administration, dietary or therapeutic iodine contamination was ruled out based on thorough history. Urinary iodine testing was not performed.

Patients with clinically suspected or documented brain, skull, or vertebral metastases (*n* = 10) received steroid prophylaxis against peritumor edema and related neurological symptoms. The steroid regimen was oral methylprednisolone, from 16 to 48 mg/day, for at least 5 days, starting the day of the first rhTSH injection. This regimen was based on our Institute's routine scheme for patients with brain or spinal cord metastases or both undergoing EBR, and was tailored to each patient's clinical situation [17, 24].

All patients received suppressive L-thyroxin (LT4), that is, the individualized dose, ranging from 1.5–2.5 mcg/kg of body weight, necessary to achieve serum TSH < 0.1 mU/L, throughout the study. In conformity with the Slovenian Radiation Protection Law, patients were hospitalized in the radionuclide therapy ward with full radiation protection from the day of radioiodine administration to the day the radiation dose rate at one meter reached <32 microSv/h. Adverse effects were evaluated through careful observation by specially trained radiation ward medical personnel, physical examination, thorough history and spontaneous patient report.

### 2.3. Assessment of Radioiodine Uptake and Outcome of rhTSH-Aided RIT

Radioiodine uptake was assessed by posttherapeutic WBS (rxWBS) and when indicated, by “spot imaging” of regions of interest, performed on the 3rd to 6th day after radioiodine administration, using a dual-headed gamma camera (Elscint, Haifa, Israel) equipped with parallel-hole, high-energy collimators. rxWBS was taken over approximately 15 minutes, with a camera velocity of 18–20 cm/min. At least 600 KCnts were acquired per “spot image”. 

In this patient group, follow-up visits were scheduled every 6–12 months, in some cases less, taking into account the clinical status and progression risk and always comprised measurement of serum Tg, TSH, thyroid hormone and anti-Tg antibody levels. Two patients also received rhTSH-aided follow-up diagnostic WBS (dxWBS), which was acquired over at least 35 minutes, with a camera velocity of 6–8 cm/min. All WBS were interpreted by experienced nuclear medicine physicians. 

The efficacy and outcome of initial rhTSH-aided RIT were assessed via a within-patient comparison of the number, location, and extent of 131-I-avid lesions on the rhTSH-aided rxWBS versus on the most recent available THW-aided rxWBS. For the second and subsequent rhTSH-aided RITs, the rhTSH-aided rxWBS was compared with the most recent prior rhTSH-aided rxWBS. At each follow-up visit a detailed history, clinical status, laboratory tests, neck ultrasonography and chest and/or bone radiography were obtained. Whenever because of tumor progression or suspicion of complications other treatment options (surgery or radiotherapy) were considered, also computed tomography, magnetic resonance imaging (MRI), or both were performed. In none of our patients PET-CT was performed. The overall outcome of the rhTSH-aided RIT(s) was assessed based on the comparison of clinical and radiological findings and serum Tg levels before the first rhTSH-aided RIT versus after the most recent rhTSH-aided RIT. All comparisons were unblinded.

Outcome categories were defined as defined by Jarzab et al. [18].

**(i) Complete response (CR):**

no pathologic uptake on WBS and full tumor remission on radiological examination, serum Tg level <1 mcg/L on LT4 therapy or <2 microg/L after rhTSH stimulation or THW;

**(ii) Partial response (PR):**

>25% decrease in tumor size and/or in serum Tg level if there was no increase in tumor size;

**(iii) Stable disease (SD):**

*≤*25% decrease to 25% increase in tumor size and/or serum Tg level;

**(iv) Progressive disease (PD):**

>25% increase in tumor size and/or in serum Tg level and/or new 131-I-avid foci on WBS.

### 2.4. Biochemical Evaluation

To confirm adequate TSH suppression and rule out LT4 over dosage and to permit the accurate use of serum Tg as a tumor marker, TSH, free LT4, free triiodothyronine (FT3), Tg, and anti-Tg-antibody (TgAb) values were determined before the first rhTSH injection, before the first injection in each subsequent rhTSH course, and 24 hr after the second injection in each rhTSH course. Effect of rhTSH RIT on Tg concentration was determined by comparison of Tg concentration before the first rhTSH injection of each RIT and two months thereafter. Serum TSH, FT3, free LT4, and Tg were measured using commercially available kits (LIAISON, DiaSorin, Saluggia, Italy). The serum TSH and Tg measurements were carried out by two-site immunoluminometric assay.

### 2.5. Statistical Methods

For this observational, open-label, nonrandomized study, only descriptive statistics were generated, with one exception. We used the Student's *t*-test, performed on Microsoft Office Excell 2003 to compare serum TSH levels after the first rhTSH course versus after the most recent THW for which data were available.

## 3. Results

### 3.1. Treatments

Our 18 patients received a total of 44 rhTSH-aided RITs: five patients received one, six had 2, two patients had 3, four had 4, and one patient had 5 rhTSH-aided RITs. The median time between rhTSH-aided RITs was 10 months (range from 6 to 22).The average cumulative rhTSH-aided RIT activity was 15.77 GBq (median: 13.75 GBq, range, 5.22 to 34.52 GBq).

### 3.2. Radioiodine Uptake

At referral for the 1st rhTSH-aided RIT all patients had proven functional cancer foci, confirmed by visible radioiodine uptake on the most recent THW-aided rxWBS (*n* = 18) or dxWBS (*n* = 2). In all patients, every rxWBS was positive, showing at least one 131-I-avid lesion; altogether 60 iodine-avid foci were detected in the 44 rxWBS.

### 3.3. Tg Response, Outcome of RhTSH-Aided RIT, and Survival

The most prominent change from basal Tg value to the 2nd month check-up Tg value was seen in patient 1 (91%), The highest rise from baseline Tg value was seen in patient 4 (3593%). Changes of Tg levels from up to at least 6 months in all patients were consistent with the results of post-RIT WBS and the other follow-up imaging modalities. 

**Figure 3:**
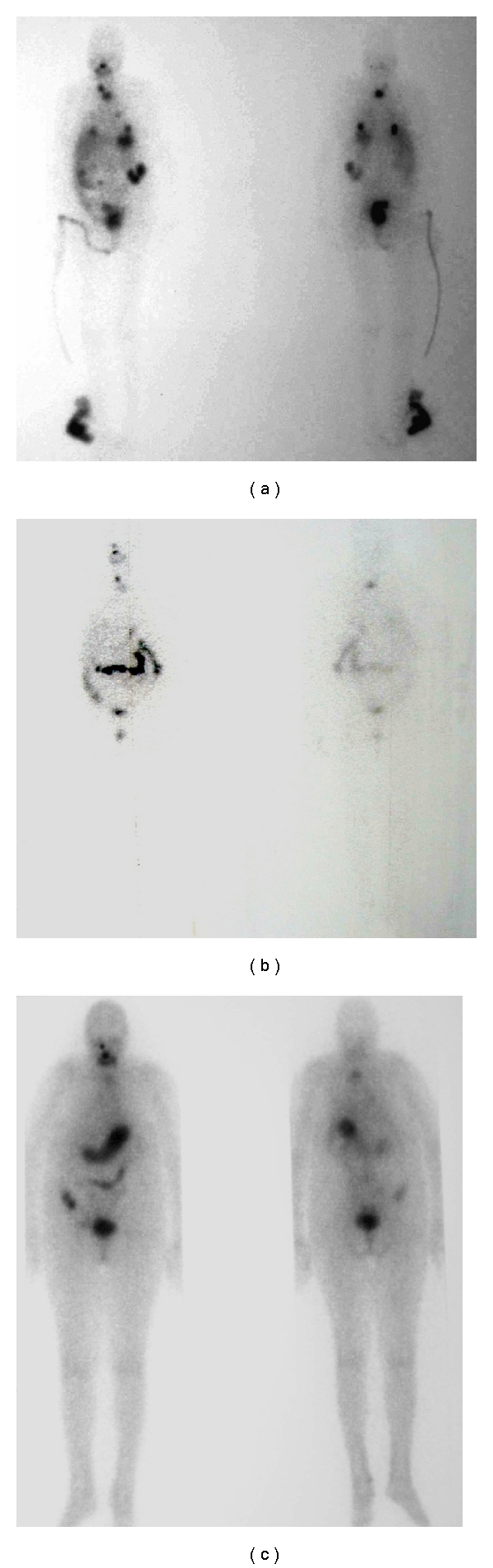
Treatment of a 73-year-old patient with Hürthle cell carcinoma. (a) rxWBS taken 48 hrs after the first application of 5.5 GBq of 131-I THW-aided RIT in 1997: pathologic uptake is visible in the thyroid bed, in the 4th thoracic vertebra (Th4), and in the lungs bilaterally. (b) An rxWBS (following a fifth RIT after hormone withdrawal with 7.4 GBq of 131-I in 2001) shows regression of metastatic disease: pathologic uptake in the lungs is no longer visible; the foci of uptake in Th4 appear to be smaller and less intense; cumulative uptake of RAI is 2%. (c) An rxWBS (following a second rhTSH-aided RIT with 7.4 GBq of 131-I in 2002) shows regression of metastatic disease: the foci of uptake in Th4 appear to be smaller and less intense; cumulative uptake of RAI is 0.05%.

No CR was observed. A PR was achieved in three (17%) patients (nos. 1, 2, 9), in whom serum Tg after initial progressive rise in Tg level at first rhTSH-aided RIT decreased 24% (minimally) and 87% (maximally) from pre-rh TSH-aided RIT level at last checkup after 2 and 6 months after last rhTSH-aided RIT. One of these three patients (patient #2) had a long-lasting response to RIT after hormone withdrawal and rhTSH-aided RIT. In this individual, a 73-year-old female with Hürthle cell carcinoma, serum Tg decreased by 35% at the first checkup at two months after the first rhTSH-aided RIT and by another 24% at the check-up at two months after the second rhTSH-aided RIT. Thyroid carcinoma remained stable for 81 months and she died because of causes not related to thyroid carcinoma. Scintigraphically and radiographically, this patient showed a complete regression of the 131-I-avid metastases in both lungs and a stabilization of the metastatic disease in the thoracic vertebra (Figure 3).

A SD was achieved in four (22%) patients (nos. 13, 14, 17 and 18), in whom serum Tg values changed from 24% decrease to 23% increase. The smallest change was observed in patient 17, where after 13% increase after first rh TSH-aided RIT steady plateau of approximately 10% decrease was observed. 

In most patients (*n* = 11, 61%) disease progressed (DP) despite rhTSH-aided RITs. The highest increase in Tg values was seen in patient 4, where the last Tg value at the last 6 month checkup after the last rhTSH-aided RIT increased for approximately 3500% compared to the value at the 2 month checkup after the last rhTSH-aided RIT despite occasional drop in Tg values at preceding rhTSH-aided RITs.

With a median 50 months of follow-up, six (33%) patients were alive. Nine (50%) patients died during follow-up because of distant metastasis 15, 16, 24, 27, 28, 33, 46, 54 and 65 (median 28) months after the first rhTSH-aided RIT. Three patients died of causes not related to thyroid cancer.

### 3.4. Other Biochemical Effects of RhTSH

Serum TSH levels after the second rhTSH injection were 58–>10000 mU/L (median, 135 mU/L). Following the rhTSH injections most patients had stable normal serum FT3 and normal to slightly elevated free FT4.

### 3.5. Side Effects of RhTSH-Aided RIT

RhTSH was in all but one patient generally well tolerated, with transient (up to five days) and mostly mild side effects: nausea in nine (50%) patients, headache in six (33%) patients, slight to moderate escalation in bone metastasis pain that was manageable with nonopioid medication in three patients, a “flu-like syndrome” or muscle cramps in two patients, and sweating in one individual.

**Figure 4:**
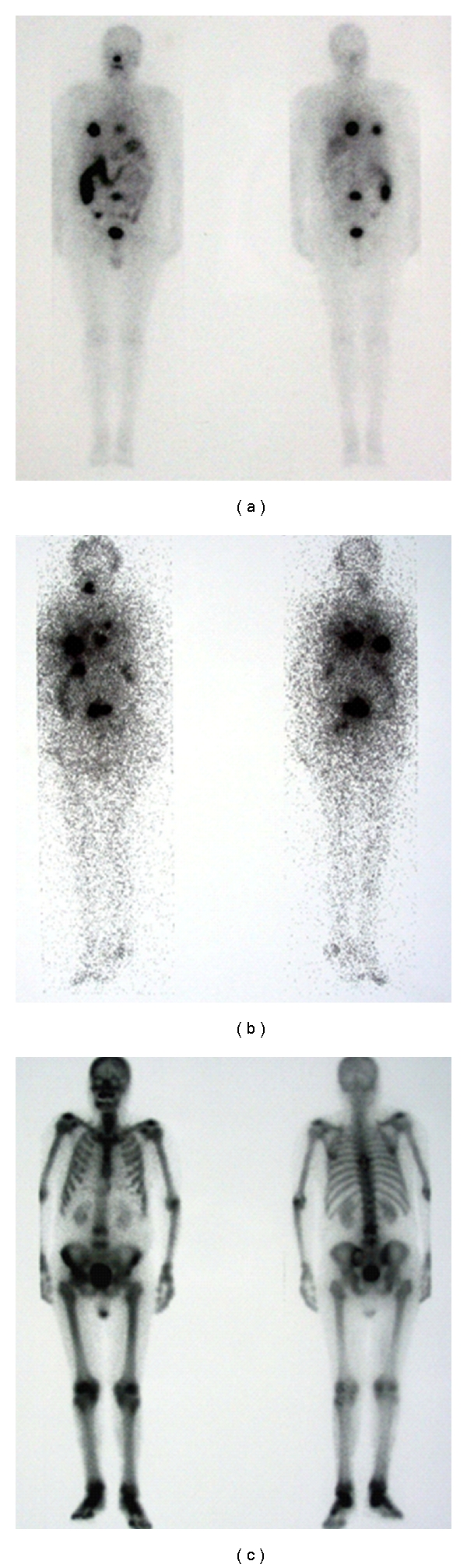
A 58-year-old patient with papillary thyroid carcinoma and poorly differentiated carcinoma. (a) An rxWBS taken 48 hrs after application of 5.5 GBq of 131-I under THW, in 2004: pathologic uptake is seen in the mid-thoracic vertebrae, throughout the right hemithorax, in 2 foci in the anterior left hemithorax, and in the lower lumbar vertebrae. (b) One year later: an rxWBS taken 48 hours after an rhTSH-aided RIT with 5.5 GBq of 131-I in the same patient, demonstrates progressive disease despite a total 9 of RITs (3 rhTSH-aided) with a cumulative activity of 49.6 GBq, EBR, and chemotherapy: pathologic uptake in the thoracic and lumbar vertebrae, as well as the bilateral pathologic accumulation in the thorax are larger and more intense, new foci of pathological uptake are seen in the right side of the neck, in the third lumbar vertebra, and faintly in the left pelvis. (c) A bone scan of the same patient, performed 4 months after the last rhTSH-aided 131-I treatment: osteolytic lesions are clearly visible in the left sacroiliac joint, and in the Th6–Th8 and L3–L5 vertebral segments. Additionally, faint osteolytic lesions may be suspected in the 5th right rib anteriorly and in the L1 and S1 vertebra.

Only one patient (no. 16) experienced serious side effects of rhTSH. This 58-year-old male with poorly differentiated papillary histology and metastases in the lungs, ribs, thoracic, and lumbar vertebrae and pelvis (Figure 4), had been heavily pretreated (6 THW-aided RITs with a cumulative 131-I activity of 32.93 GBq, EBR, and chemotherapy). The patient received steroid prophylaxis with methylprednisolone 56 mg/day. After the first injection of his 2nd course of rhTSH, he developed severe vomiting, which subsided after a larger dose of methylprednisolone (120 mg/day). He also suffered severe pain in his known bone metastatic sites, that despite high-dose steroid therapy escalated after the second rhTSH injection to become manageable only by opioid medication. Three days after the second rhTSH injection (i.e., 2 days after administration of 7.2 GBq of radioiodine), a spastic paraparesis occurred. Despite anti-edematous therapy with 10% mannitol, furosemide, and continued high-dose steroids, the paraparesis progressed within hours to a complete spastic paraplegia due to a pathological 7th thoracic vertebra fracture and spinal cord compression that were confirmed by an MRI. The paraplegia required immediate surgical decompression, which was feasible, since the patient's radiation dose rate was already below the regulatory limit. At the time of surgical procedure the radiation dose rate of this patient was below 32 microSv/hour. None of the surgical team personnel or nursing staff which took care of this patient during or after surgical procedure reached the monthly or annual receivable dose (regulatory limit in Slovenia is 20 mSV/year). We did not expect such a detrimental course, especially for the first rhTSH-aided RIT ended well. It is possible that neurological symptoms were triggered by rhTSH-aided RIT. 

In another patient with a previously known supraventricular tachycardia, an episode of atrial fibrillation with rapid ventricular response was noted after the second rhTSH injection of the third course of rhTSH, most likely caused by accompanying fever due to rhTSH injection and was not accompanied by thyrotoxicosis. The patient in question also suffered a flu-like syndrome due to rhTSH injection and a headache, for laboratory parametra excluded infectious cause of a fever. Fibrillation lasted for a day and came to an end after antipyretic therapy was started. This adverse effect of moderate grade was observed before the radioiodine administration on day three, which caused no change in the intensity of the adverse events.

## 4. Discussion

Our group of patients given rhTSH-aided RIT for metastatic DTC represents, to our knowledge, the second largest such published series for whom outcomes of this modality have been reported. Our patients had a variety of unfavorable prognostic characteristics—72% were over 65 and 61%, over 70 years old, all had distant metastases, 56% in the bone, half of whom also had soft tissue metastases, and 50% had Hürthle cell or poorly differentiated papillary or follicular histology. Explaining the general frailty of our series, our most important inclusion criterion was inability to undergo THW, because of one or more of advanced age, serious concomitant illness, late-stage DTC, or history of severe hypothyroid symptoms or of tumor progression during THW. Beyond this, our patients were heavily pretreated, 94% having received THW-aided RIT (median, 5 treatments, mean cumulative activity, 27.89 GBq), 67%, EBR, and 44%, chemotherapy. 

Despite the “unfavorable profile” of our patient population, seven (39%) of our 18 patients had results suggesting some therapeutic benefit of rhTSH-aided RIT, ranging from transient disease stabilization during rhTSH-aided RITs, disease progression after first few rhTSH-aided RITs with a sudden drop to a PR, and improvement after each rhTSH-aided RITs with disease progression between them, that included regression of lung and reduction of bone metastases. In a period of PR and SD, most of the patients had less problems related to DTC compared to period of PD. However, unsurprisingly given the characteristics of our series, 61% of our patients had PD despite one or more rhTSH-aided RITs, and 66% died during the follow-up, nine (50%) because of progression of distant metastases.

Compared to other published series [8, 17–19, 25–27], our clinical benefit rate (39%) falls into the low end (40%–73%), and our mortality rate probably because of long follow-up period, into the high end of the spectrum (0%–33%). However, our patient population appears to have been older, to have included a higher proportion of patients with bone metastases, unfavorable histology, or both, and to have been more heavily pretreated than most of the other published series. Moreover, comparisons must be made with caution because of the small numbers of most series and disparate response criteria and length of follow-up in different studies. 

Notwithstanding the advanced age, frailty, and heavy tumor burden of our patients, rhTSH was well tolerated, but one of our patients had serios side effects (no. 16). Despite steroid prophylaxis, very shortly after his rhTSH injections, he suffered severe bone pain escalation and vomiting and a pathological fracture of the 7th thoracic vertebra with spinal cord compression resulting in spastic paraplegia requiring immediate surgical decompression. This case was similar to isolated cases reported elsewhere [12–19]. The patient's lack of response to high-dose steroid prophylaxis and treatment and documented rapid tumor growth after previous THW-aided RIT suggest that this adverse event might have been the consequence not just of tumor edema, but growth after rhTSH. However, another patient in our series (no. 13) with similar disease characteristics and premedication had no clinical evidence of tumor expansion after three courses of rhTSH and achieved at least transient disease stabilization. Moreover, none of our patients with neck, mediastinal, or pulmonary metastases experienced dyspnea or a choking feeling that could be associated with tumor edema, and six of ten patients with bone metastases reported no, and three of ten only mild escalation of skeletal pain after rhTSH, less intensity and duration than experienced with previous THW. Therefore we share the opinion [25] that even in patients with known or suspected metastases in confined spaces, rhTSH application for RIT usually does not present a notable risk if special caution and steroid coverage are employed.

The present study confirms and extends our earlier findings [20, 28] that patients with histologically confirmed Hürthle cell carcinoma frequently exhibit uptake and sometimes derive clinical benefit, from large radioiodine activities. Numerous 131-I-avid lesions were detected in five of six of our patients with such histology, and our patient with the longest and most radiologically extensive response belonged to this subgroup. The only other previously published Hürthle cell carcinoma patient given rhTSH-aided RIT also had radioiodine uptake on his posttherapy scan, but appears not to have responded to this treatment [19].

Our study had several important limitations. First, its design was observational and prospective only for the rhTSH-aided RIT component of the patients' overall therapy, and follow-up was relatively short. In addition, all comparisons and observations were unblinded. Second, since we did not conduct dosimetry or empirically adjust the radioiodine activity for the faster 131-I clearance under euthyroid conditions [23] we may have used a suboptimal radioiodine activity. Our reasons for omitting dosimetry included the well-known limitations of this maneuver [11, 29], the wish to spare frail patients an additional procedure, and the additional cost of rhTSH for this procedure. Moreover, we note that to our knowledge only one published study [20] adjusted the radioiodine activity for rhTSH versus THW.

Another limitation is that the heterogeneity in the number of courses and cumulative activities of rhTSH-aided RIT, and particularly in the amount and recent prior THW-aided RIT, EBR, and chemotherapy, make it difficult to gauge the degree to which the PRs or disease stabilizations are attributable solely to rhTSH-aided RIT. For example, all but two of the six patients with clinical benefit from rhTSH-aided RIT had EBR with or without chemotherapy within 1–3 months before the first rhTSH-aided RIT. However, the clinical benefit would appear to be attributable mainly or solely to rhTSH-aided RIT in our long-standing partial responder (patient 2) and in one patient with SD (no. 17), who had long intervals (27 and 9 months, respectively, since prior therapy.

An open issue is whether to continue RIT in heavily treated patients with aggressive but functional metastases, in whom the modality achieves only transient disease stabilization. In our institution we present each such patient to a team of doctors, who take part in treating thyroid carcinoma patients (including surgeons, who are pro-rhTSH-aided RIT, mostly because they believe it is last possible treatment), and the decision whether to continue with rhTSH-aided RITs depend on the decision of the team mentioned. Mostly we try to continue with rhTSH-aided RITS as long as posible, despite the pretreatment with RIT, as long as there is the smallest possibility of success. Sometimes the disease stabilization means important clinical palliation. Consideration should be given to enrolling such patients in clinical trials of molecular approaches, when feasible.

Recently, additional outcome data from the Institute Goustave-Roussy (IGR), Villjuif, France, suggests that in the distant metastatic DTC setting, RIT is most effective in younger patients with small, functional soft tissue lesions [30]. These data also suggest that >95% of CRs to RIT can be attained by fractionated administration of up to 27 GBq of 131-I. Data of our and other studies [7, 17, 19, 20, 25, 27, 29, 30] prove that rhTSH-aided RIT in the late-stage metastatic DTC is safe and may be effective. RhTSH-aided RIT improve patient quality of life and ability to work [4–6, 14] and decrease radiation exposure in extrathyroidal compartments [16]. A comparative trial of rhTSH versus THW as an adjunct to RIT may be highly clinically relevant, and feasible, in these earlier-stage patients.

In conclusion, use of rhTSH offers the possibility of high-dose RIT to patients with metastatic DTC who otherwise would be unable to receive such treatment due to the risk of intolerable or serious adverse reactions to the clinical hypothyroidism of THW. RhTSH-aided RIT has minimal side effects in most patients and offers therapeutic benefit even in some patients with late-stage DTC.

## Figures and Tables

**Table 1:** Patients treated with rhTSH-aided radioiodine therapy.

Patient number	Age (years)	Gender	Tumor histology	Tumor stage at initial diagnosis	Primary surgical treatment	Prior THW-aided 131-I (GBq^†^)	Metastatic site(s) at time of first rhTSH-aided RIT	Previous EBR and/or chemotherapy (ChT)	Indication(s) for rhTSH	Number of rhTSH-aided 131-I (GBq)	Clinical effect of rhTSH-aided 131-I	Outcome	Survival after the first rhTSH-aided 131-I (months)
1	81	F	Fo	T4N0M0	Total thyroidectomy	38.55	Bone	EBR	Advanced age, potential compressive neurological symptoms	2	1×PR 1×PD	Dead of other reasons	22
2	73	F	H	T4N0M1	Total thyroidectomy	27.89	Bone, lung	EBR, ChT	Progressive paraparesis, potential additional compressive neurological and respiratory symptoms	2	2×PR	Dead of other reasons	81
3	79	F	H	T4N0M0	Total thyroidectomy	39.47	Lung	EBR	Advanced age, potential respiratory symptoms, concomitant diseases	1	1×PD	Dead of metastatic disease	15
4	63	F	H	T4N0M1	Total thyroidectomy	15.65	Lung	EBR, ChT	Potential respiratory symptoms	5	4×PD 1×SD	Dead of metastatic disease	54
5	72	F	P, FV	T4N0M1	Total thyroidectomy	68.33	Bone	EBR, ChT	Advanced age, potential compressive neurological symptoms	3	3×PD	Alive	99
6	71	M	H	T3N0M0	Subtotal thyroidectomy	50.32	Bone, lung	EBR, ChT	Advanced age, potential compressive neurological and respiratory symptoms	2	2×PD	Dead of metastatic disease	16
7	72	F	Fo, INS	T4N0M1	Total thyroidectomy	29.60	Bone	EBR, ChT	History of severe hypothyroid symptoms, hypertension, history of breast carcinoma	4	4×PD	Dead of metastatic disease	65
8	69	F	P, FV	T4N0M0	Total thyroidectomy	36.04	Bone, lung	EBR	Heart disease, potential compressive neurological and respiratory symptoms, history of severe hypothyroid symptoms	2	2×PD	Dead of metastatic disease	33
9	78	F	H	T4N1M1	Total thyroidectomy	14.73	Lung, right kidney	—	Advanced age, potential respiratory symptoms	4	1×PD 2×SD 1×PD	Alive	91
10	62	M	P, FV	T4N1M0	Total thyroidectomy and modified RND	22.64	Lung	EBR	Potential respiratory symptoms	2	2×PD	Dead of metastatic disease	46
11	82	F	H	T3N0M0	Total thyroidectomy	3.88	Mediastinum	EBR	Advanced age, history of severe hypothyroid symptoms	2	1×PD	Alive	67
12	79	F	Fo	T3N0M0	Total thyroidectomy	21.46	Bone, mediastinum, central neck compartment	EBR	Advanced age, potential compressive neurological symptoms	1	1×PD	Dead of metastatic disease	28
13	66	F	Fo, PoD	T2N0M1	Total thyroidectomy	5.69	Bone, brain	EBR, ChT	Potential neurological symptoms from metastases	4	4×SD	Dead of metastatic disease	24
14	77	F	Fo	T3N0M0	Total thyroidectomy	3.58	Lung	—	Advanced age, heart disease	3	3×SD	Alive	81
15	64	M	Fo	T4N0M0	Total thyroidectomy	0	Mediastinum, central neck compartment	EBR	Heart disease, etilic hepatopathy, potential compressive respiratory symptoms	1	1×PD	Dead of other reasons	22
16	58	M	P, PoD	T4N0M0	Total thyroidectomy and prophylactic mRND	32.93	Bone, lung	EBR	Potential compressive neurological and respiratory symptoms	2	2×PD	Dead of metastatic disease	27
17	37	M	P	T4aN1bM1	Total thyroidectomy and modified RND	3.70	Lung	EBR, ChT	Potential respiratory symptoms	4	4×SD	Alive	74
18	83	M	Fo	T3N0M1	Total thyroidectomy	35.00	Bone, liver	—	Advanced age, potential compressive neurological symptoms, concomitant diseases	1	1×SD	Alive	61

F: female; Fo: follicular carcinoma; FV: follicular variant; H: Hürthle cell carcinoma; INS: insular variant; M: male; P: papillary carcinoma; PoD: poorly differentiated; RIT: radioiodine treatment; ChT: chemotherapy, EBR: external beam radiotherapy; rhTSH: recombinant human thyroid-stimulating hormone; THW: thyroid hormone withdrawal.

*UICC: International Union Against Cancer, seventh edition, 2009.

^†^ Includes remnant ablation.
